# High-sensitive and fast response to 255 nm deep-UV light of CH_3_NH_3_PbX_3_ (X = Cl, Br, I) bulk crystals

**DOI:** 10.1098/rsos.180905

**Published:** 2018-09-05

**Authors:** Zhaojun Zhang, Wei Zheng, Richeng Lin, Feng Huang

**Affiliations:** 1 School of Materials, Sun Yat-Sen University, Xingang Xi Road No. 135, Guangzhou 510275, People's Republic of China; 2 School of Physics, Sun Yat-Sen University, Xingang Xi Road No. 135, Guangzhou 510275, People's Republic of China; 3 State Key laboratory of Optoelectronic Materials and Technologies, Sun Yat-Sen University, Xingang Xi Road No. 135, Guangzhou 510275, People's Republic of China

**Keywords:** CH_3_NH_3_PbX_3_, bulk crystal, deep-UV detection

## Abstract

Deep-UV light detection has important application in surveillance and homeland security regions. CH_3_NH_3_PbX_3_ (X = Cl, Br, I) materials have outstanding optical absorption and electronic transport properties suitable for obtaining excellent deep-UV photoresponse. In this work, we have grown high-quality CH_3_NH_3_PbX_3_ (X = Cl, Br, I) bulk crystals and used them to fabricate photodetectors. We found that they all have high-sensitive and fast-speed response to 255 nm deep-UV light. Their responsivities are 10–10^3^ times higher than MgZnO and Ga_2_O_3_ detectors, and their response speeds are 10^3^ times faster than Ga_2_O_3_ and ZnO detectors. These results indicate a new promising route for deep-UV detection.

## 1. Introduction

The increasingly irreplaceable application of deep-ultraviolet (deep-UV: 200–280 nm) technology (imagery, warning and secure communication) in surveillance, homeland security and civil regions, makes the high-sensitive and fast-speed deep-UV detectors being urgently demanded [1–5]. Compared to cumbersome vacuum phototube detectors, semiconductor ones are lightweight, robust and have low operating voltage [6,7]. There are generally two detection strategies for semiconductor-based deep-UV detectors. One approach is to use wide bandgap semiconductors such as AlGaN, MgZnO, Ga_2_O_3_ or diamond [8–12]. However, the high-temperature and complex growth condition make it difficult to obtain high-quality materials, and thus the performance of the fabricated detectors are always far from expected [13]; another alternative approach is employing narrow band-gap Si diode detectors equipped with UV-pass filters [14,15]. However, the deep-UV detection of Si diode is still barely satisfactory, as the large absorption of deep-UV light of Si makes it difficult for the photo-generated carrier to reach the depletion layer. Therefore, it is still urgently needed to explore new semiconductor materials which have both facile growth method and excellent deep-UV response performance.

Recently, organic–inorganic perovskite CH_3_NH_3_PbX_3_ (X = Cl, Br, I) have attracted intensive attention in solar cells, luminescence, photodetection etc. [16–19]. High-crystalline quality CH_3_NH_3_PbX_3_ crystals can be easily grown using simple low-temperature (less than 100°C) solution method [20–22]. And they have large absorption coefficient of approximately 10^5^ cm^−1^ in deep-UV spectral range [17,23,24], high carrier mobility even exceeds 100 cm^2^ V^−1^ s^−1^) [25] and long carrier transport length up to hundreds of micrometres [18,26], which make them promising for showing high-sensitive and fast-speed deep-UV response performance. Several studies have reported the photodetection properties of CH_3_NH_3_PbX_3_ [19,27,28], which generally concern visible light or radiation detection. Special comprehensive research on their deep-UV detection performance has not been reported. As mentioned above, narrow band-gap semiconductor also has application possibilities in deep-UV detection with the aid of UV-pass filters. Therefore, studies on deep-UV detection performance of CH_3_NH_3_PbX_3_ have practical significance.

In this work, we have grown high-quality bulk CH_3_NH_3_PbX_3_ crystals and used them to fabricate photodetectors. The deep-UV detection performance of CH_3_NH_3_PbCl_3_, CH_3_NH_3_PbBr_3_ and CH_3_NH_3_PbI_3_ were comprehensively studied. Under illumination of 1.5 mW cm^−2^ 255 nm light and 5 V bias, CH_3_NH_3_PbCl_3_, CH_3_NH_3_PbBr_3_ and CH_3_NH_3_PbI_3_ respectively show responsivities of approximately 450, 300 and 120 mA W^−1^, and rise time of 15, 2.5 and 2 ms. These results manifest that CH_3_NH_3_PbX_3_ are promising candidates for deep-UV detection.

## 2. Material and methods

**Figure 1.**
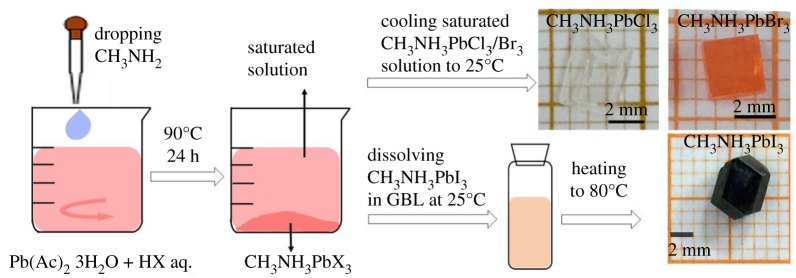
Schematic representation of powder synthesis and single crystals growth of CH_3_NH_3_PbX_3_ (X = Cl, Br, I). CH_3_NH_3_PbX_3_ powders are synthesized through reaction between Pb(Ac)_2_, CH_3_NH_2_ and HX aqueous solution. CH_3_NH_3_PbCl_3_/Br_3_ crystals are grown by cooling the saturated precursor solution from 90°C to 25°C. CH_3_NH_3_PbI_3_ crystals are grown from the saturated solution of CH_3_NH_3_PbI_3_ powder in γ-butyrolactone (GBL) by gradually heating (from 25°C to 80°C) due to its negative solubility temperature coefficient.

CH_3_NH_3_PbX_3_ powders were synthesized from halogen acid aqueous solution using the method illustrated in figure 1. Firstly, 5 g lead(II) acetate trihydrate (AR) was dissolved in 20 ml HCl/HBr/HI acid solution in a 50 ml flask under rigorous stirring. Then PbCl_2_/PbBr_2_/PbI_2_ powders are generated in the flask. Secondly, 10 ml methylamine (40% wt/wt aq. sol.) was added to the above blend solution and CH_3_NH_3_PbX_3_ powders precipitated. It should be noted that the addition of methylamine should be drop by drop under rigorous stirring because the reaction is violently exothermic. Once the CH_3_NH_3_PbCl_3_/Br_3_ powder was obtained, keeping the blend solution at 90°C and under rigorous stirring for 24 h until the powder was fully dissolved. Then stopping stirring and absorbing the supernatant liquid and placed them into a 20 ml serum bottle. Then slowly cooling this saturated precursor solution to room temperature, 1–3 mm^3^ CH_3_NH_3_PbCl_3_/Br_3_ crystals were obtained. Yellow needle-like CH_3_NH_3_PbI_3_ · nH_2_O crystals are formed when CH_3_NH_3_PbI_3_ encounters water below 50° [29]. Thus, CH_3_NH_3_PbI_3_ single crystals were grown from organic solvent γ-butyrolactone using inverse temperature crystallization (ITC) method due to its negative solubility temperature coefficient. By fully dissolving the CH_3_NH_3_PbI_3_ powder in γ-butyrolactone (approx. 0.3 g ml^−1^) and slowly heating the solution from room temperature to 80°C with a rate of approximately 5°C h^−1^, 5 mm^3^ CH_3_NH_3_PbI_3_ crystals can be obtained.

Au films were deposited on the crystal surface as electrodes by thermal evaporation. The photo-response performance was measured using self-built system with 255 nm LED as light source.

## 3. Results and discussion

**Figure 2.**
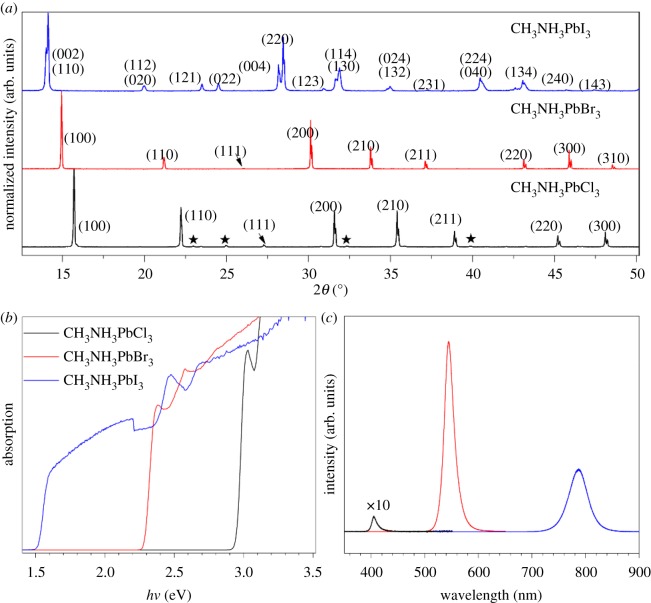
(*a*) Powder X-ray diffraction (XRD) patterns of the three crystals. CH_3_NH_3_PbCl_3_ and CH_3_NH_3_PbBr_3_ both belong to cubic phases, and CH_3_NH_3_PbI_3_ belongs to tetragonal phase. (*b*) The dependence of absorption of CH_3_NH_3_PbX_3_ on the photon energy. (*c*) Photoluminescence spectra of CH_3_NH_3_PbX_3_ crystals excited by 325 nm laser. For clarity, the photoluminescence intensity of CH_3_NH_3_PbCl_3_ was multiplied by 10 times.

The powder XRD patterns of CH_3_NH_3_PbX_3_ crystals are shown in figure 2*a*, which agree well with previously reported results [18,20,21]. The residual weak peaks denoted by stars in the pattern of CH_3_NH_3_PbCl_3_ come from PbCl_2_. XRD patterns of CH_3_NH_3_PbCl_3_ and CH_3_NH_3_PbBr_3_ are very close because they both belong to cubic system (space group of Pm-3m) with different lattice constants of 5.67 Å for CH_3_NH_3_PbCl_3_ and 5.92 Å for CH_3_NH_3_PbBr_3_. CH_3_NH_3_PbI_3_ belongs to tetragonal phase (space group I4/m) with lattice constants of *a* = *b* = 8.83 Å and *c* = 12.69 Å. Their different crystal structures resulted from the different ion radius of Cl (1.81 Å), Br (1.96 Å) and I (2.2 Å). The large ion radius of I makes CH_3_NH_3_PbI_3_ distort from cubic to tetragonal phase.

Furthermore, detailed optical properties of CH_3_NH_3_PbX_3_ crystals were also studied comprehensively. Steady state UV-Vis diffuse reflection spectra of CH_3_NH_3_PbX_3_ powder were collected. According to Kubelka–Munk function, the dependence of (*F*(*R*∞)*hv*)^2^ on photon energy is given in figure 2*b*. As can be seen, sharp band edges are clearly observed, indicating the direct bandgaps of CH_3_NH_3_PbX_3_. Relying on estimation from Tauc/Davis–Mott model [30,31], through extrapolating the linear range of (*F*(*R*∞)*hv*)^2^ to photon energy (*hv*) intercept, the bandgaps of CH_3_NH_3_PbCl_3_, CH_3_NH_3_PbBr_3_ and CH_3_NH_3_PbI_3_ are estimated to be 2.95, 2.28 and 1.52 eV, respectively. The gradual lowering of CH_3_NH_3_PbX_3_ bandgap with halogen changing from Cl to Br to I is mainly ascribed to the lowering valence band maximum formed by halogen orbitals from 3p to 4p to 5p [32]. Room temperature photoluminescence spectra of CH_3_NH_3_PbX_3_ single crystals are displayed in figure 2*c*, and the strong band–band emission indicates the high crystalline quality of CH_3_NH_3_PbX_3_ single crystals. Luminescence peak positions show a gradual red-shift from 405 nm for CH_3_NH_3_PbCl_3_ to 545 nm for CH_3_NH_3_PbBr_3_ to 787 nm for CH_3_NH_3_PbI_3_, which are ascribed to their different bandgaps. Compared to previously reported CH_3_NH_3_PbCl_3_ which has no emission at room temperature [24], the observed emission in our CH_3_NH_3_PbCl_3_ crystal indicates its high crystalline quality. The luminescence intensity of CH_3_NH_3_PbBr_3_ and CH_3_NH_3_PbI_3_ are even 10^2^ times higher than that of CH_3_NH_3_PbCl_3_ under the same measurement condition, which are attributed to their different band structures, exciton energies and carrier lifetimes.

**Figure 3.**
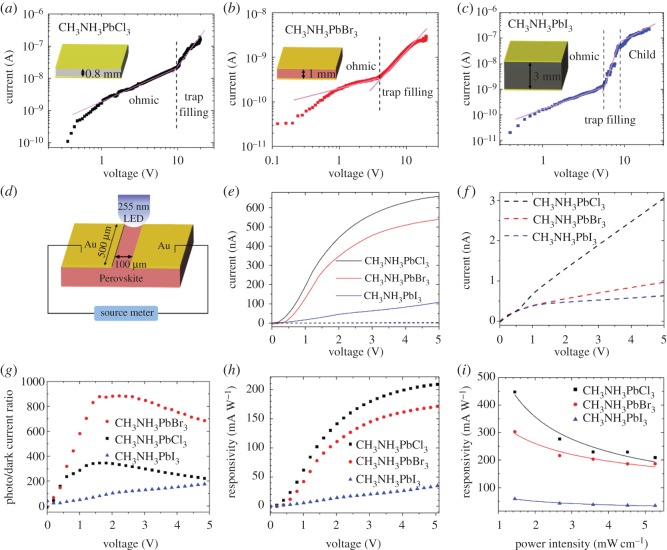
Current versus voltage in logarithmic coordinates (log*I*–log*V*) under dark condition of sandwich structure (*a*) CH_3_NH_3_PbCl_3_, (*b*) CH_3_NH_3_PbBr_3_, and (*c*) CH_3_NH_3_PbI_3_ devices, which show different regions marked as ohmic (*I* ∝ *V*), trap filling (*I* ∝ *V*^3^), and Child (*I* ∝ *V*^2^). The insets show the diagram of device structure. (*d*) Schematic diagram of planar MSM detectors and photoresponse measurement system. (*e*) Dark currents (dotted lines) and photocurrents (solid lines) under illumination of 5.3 mW cm^−2^ 255 nm light versus voltage of three planar MSM detectors. (*f*) Dark current versus voltage (*I*–*V*) of three planar MSM detectors. (*g*) Photo/dark current ratio and (*h*) responsivity of three CH_3_NH_3_PbX_3_ detectors with varying voltage. (*i*) The responsivity of three CH_3_NH_3_PbX_3_ detectors with increasing power intensity.

To estimate the trap densities of the three CH_3_NH_3_PbX_3_ crystals, we fabricated sandwich-type devices by depositing two Au electrodes on the top and bottom faces of the crystals. Their *I*–*V* plots of them under dark condition are shown in figure 3*a*–*c*. As seen from figure 3*a*, the dark current of CH_3_NH_3_PbCl_3_ shows linear dependence on voltage (*I* ∝ *V*) under low voltage, which belongs to ohmic region. When voltage is larger than 10.3 V, charge carriers start to occupy the trap states and the current rises sharply with increasing voltage (*I* ∝ *V^n^*, *n* > 3), which is trap-filled region. Similarly, CH_3_NH_3_PbBr_3_ and CH_3_NH_3_PbI_3_ both have such transition at about 3.9 V and 5.5 V, respectively. According to the space-charge-limited current (SCLC) model [20,21], the transition voltage (*V*_TFL_) from ohmic to trap-filled region is proportional to traps density (*n*_traps_) and the square of electrode gap (*L*) as described by the relation:

3.1 $$n_{\mathrm{traps}}=\frac{2\varepsilon \varepsilon_{0}V_{\mathrm{TFL}}}{eL^{2}}$$

dielectric constant *ɛ*(CH_3_NH_3_PbCl_3_) = 23.9, *ɛ*(CH_3_NH_3_PbBr_3_) = 25.5, *ɛ*(CH_3_NH_3_PbI_3_) = 28.8 [33], ***ɛ***_0_ denotes vacuum permittivity dielectric constant 8.85 × 10^12^ C V^−1^ m^−1^), *L* is electrode gap (equal to the crystal thickness, 0.8 mm for CH_3_NH_3_PbCl_3_, 1 mm for CH_3_NH_3_PbBr_3_ and 3 mm for CH_3_NH_3_PbI_3_) and *e* is the elementary charge *e* = 1.6 × 10^−19^ C. According to the formula (3.1), we find that *n*_traps_ (CH_3_NH_3_PbCl_3_) is estimated to be approximately 8.4 × 10^10^ cm^−3^, *n*_traps_ (CH_3_NH_3_PbBr_3_) is around approximately 2.1 × 10^9^ cm^−3^, and *n*_traps_ (CH_3_NH_3_PbI_3_) is about approximately 3.2 × 10^9^ cm^−3^.

To obtain high performance detector, carrier recombination should be decreased to the largest extent. CH_3_NH_3_PbX_3_ have been demonstrated to have large absorption coefficient of approximately 10^5^ cm^−1^ for deep-UV light [23], it is estimated that penetration depth of incident deep-UV photons is only about hundreds of nanometres. Thus, the materials for fabricating detectors should be as thin as possible on the premise of completely absorbing the incident light. The schematic diagram of the detectors is shown in figure 3*d*. The Au electrodes are connected to the outer circuit (source meter) using a four-probe station equipped with a microscope.

The *I*–*V* plots of CH_3_NH_3_PbX_3_ detectors under illumination of 5.3 mW cm^−2^ 255 nm light and dark condition are given in figure 3*e*, and the enlarged plots of *I*–*V* curves under dark condition are shown in figure 3*f*. As seen from figure 3*f*, under 5 V bias, the dark currents are approximately 3 nA for CH_3_NH_3_PbCl_3_, approximately 0.8 nA CH_3_NH_3_PbBr_3_ and approximately 0.6 nA for CH_3_NH_3_PbI_3_. When the voltage is smaller than 2.5 V, the dark current of CH_3_NH_3_PbBr_3_ is smaller than that of CH_3_NH_3_PbI_3_; when the bias is larger than 2.5 V, the result reverses. As seen from figure 3*e*, the photocurrents show gradual decrease with halogen varying from Cl to Br to I. For CH_3_NH_3_PbCl_3_ and CH_3_NH_3_PbBr_3_, photocurrents approach saturation with increasing voltage, which is attributed to phonon scattering on photo-generated carriers. Under high voltage, carriers are scattered heavily by phonons, thus the dependence of carrier drift velocity on voltage deviates from linear relation and approaches saturation, which leads to current saturation. While the photocurrent of CH_3_NH_3_PbI_3_ does not show obvious saturation within the measured voltage range, indicating that CH_3_NH_3_PbI_3_ has a higher saturation voltage than CH_3_NH_3_PbCl_3_ and CH_3_NH_3_PbBr_3_, which can be ascribed to their different intrinsic carrier concentration and phonon energy. Figure 3*g* displays the photo/dark current ratios of CH_3_NH_3_PbX_3_ detectors under increasing voltage, the maximum photo/dark current ratios are nearly 900 for CH_3_NH_3_PbCl_3_, 320 for CH_3_NH_3_PbBr_3_ and 190 for CH_3_NH_3_PbI_3_. The responsivity is defined as *R* = *I*/*AP*, where *I* represents the photocurrent, *P* is the incident light power, and *A* is the absorption area of device [34–37]. Illuminated under 255 nm light with power intensity of 5.3 mW cm^−2^, the responsivity versus voltage is shown in figure 3*h*. At 5 V voltage, the responsivities are 210 mA W^−1^ for CH_3_NH_3_PbCl_3_, 190 mA W^−1^ for CH_3_NH_3_PbBr_3_ and 40 mA W^−1^ for CH_3_NH_3_PbI_3_.

Responsivities under illumination with increasing powder intensity given in figure 3*i* show a gradual decreasing trend, indicating that the detectors operate on photoconductive effect of CH_3_NH_3_PbX_3_ elaborated as follows. Once the incident photons are absorbed, excitons are generated inside the perovskite crystals. Under the applied voltage, these excitons were dissociated to be free electrons and holes and transported to the external circuit; finally the photocurrent is measured. Under higher power intensity light illumination, the effective traps are filled, leading to the decrease of photoconductive gain and thus responsivity also decreases [38]. As seen from figure 3*i*, under illumination of 1.5 mW cm^−2^ 255 nm light, the responsivities are 450 mA W^−1^ for CH_3_NH_3_PbCl_3_, 300 mA W^−1^ for CH_3_NH_3_PbBr_3_, and 120 mA W^−1^ for CH_3_NH_3_PbI_3_, respectively. As summarized in table 1, these results are 10^1^–10^3^ times larger than previously reported wide bandgap semiconductors based deep-UV detectors such as Al*_x_*Ga_1−*x*_N (34 mA W^−1^) [8], Mg*_x_*Zn_1−*x*_O (0.1 mA W^−1^) [9], LaAlO_3_ (72 mA W^−1^) [43], Ga_2_O_3_ (0.32 mA W^−1^) [39], SrRuO_3_/BaTiO_3_/ZnO [40], ZnO-Ga_2_O_3_ (9.7 mA W^−1^) [41] and MgZnO (0.16 mA W^−1^) [42]. As another determinant of detector performance, external quantum efficiency (EQE) is defined as the number of generated electrons per incident photon. EQE equals to *Rhc/eλ*, where *h* is the Planck's constant, *c* is the velocity of light, and *λ* is the wavelength of incident light [34,44]. Illuminated under 255 nm light with power intensity of 5.23 mW cm^−2^, EQE is 219% for CH_3_NH_3_PbCl_3_, 146% for CH_3_NH_3_PbBr_3_ and 58% for CH_3_NH_3_PbI_3_, respectively. When illuminated under 1.5 mW cm^−2^ light, the EQE is 102%, 93% and 19%, respectively.

**Table 1:** Comparison of the responsivity of different semiconductor materials to deep-UV light.

material	light (nm)	bias (V)	*R* (mA W^−1^)	EQE (%)
CH_3_NH_3_PbCl_3_	255	5	450	219
CH_3_NH_3_PbBr_3_	255	5	300	146
CH_3_NH_3_PbI_3_	255	5	120	58
Al*_x_*Ga_1−*x*_N [8]	267	20	34	16
Mg*_x_*Zn_1−*x*_O [9]	250	10	0.1	0.05
Ga_2_O_3_ [39]	185	10	0.3	0.2
SrRuO_3_/BaTiO_3_/ZnO [40]	260	6	71.2	34
ZnO-Ga_2_O_3_ [41]	251	0	9.7	—
MgZnO [42]	250	0	0.16	—

Compared to CH_3_NH_3_PbBr_3_ and CH_3_NH_3_PbI_3_, CH_3_NH_3_PbCl_3_ shows higher responsivity and EQE. For photoconductive detector, trap states inside the photosensitive materials will capture the photo-generated electrons (holes) and carrier lifetime of holes (electrons) will be elongated and hence responsivity is improved [38]. Simultaneously, the captured carriers also slow the response speed, meaning that responsivity increase is always accompanied by response speed decrease.

From analysis on the dark current of three sandwich-type CH_3_NH_3_PbX_3_ devices using SCLC model, it is suggested that CH_3_NH_3_PbCl_3_ has higher density of trap states than that of CH_3_NH_3_PbBr_3_ and CH_3_NH_3_PbI_3_. If the higher density of trap states of CH_3_NH_3_PbCl_3_ leads to the higher responsivity, it will also induce the slower response speed of CH_3_NH_3_PbCl_3_ detectors. To verify this point, analysis on response speed will be given as follows.

**Figure 4.**
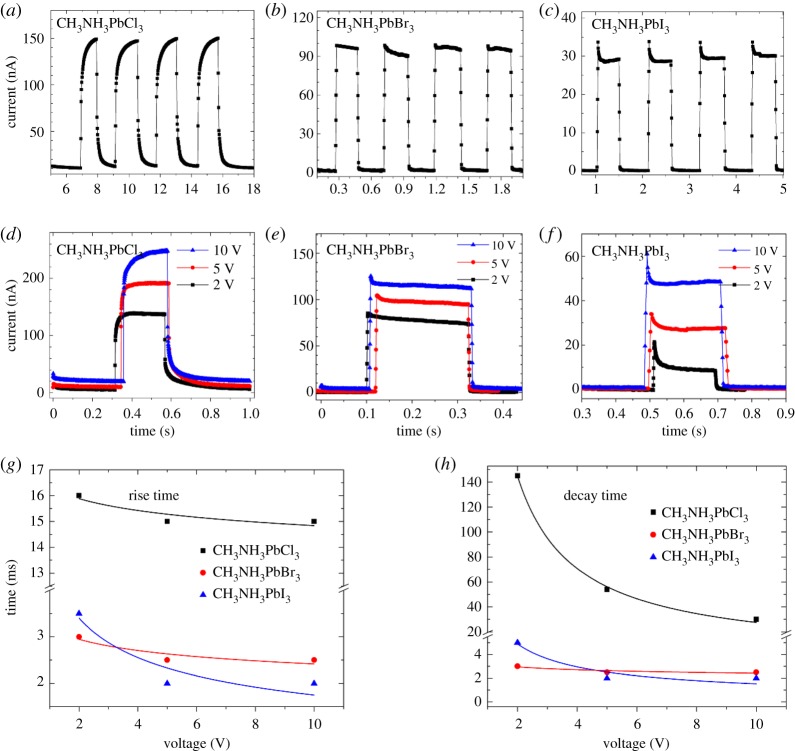
Photo-switching characteristics of (*a*,*d*) CH_3_NH_3_PbCl_3_ (*b*,*e*) CH_3_NH_3_PbBr_3_ and (*c*,*f*) CH_3_NH_3_PbI_3_ photodetectors illuminated under modulated 255 nm light. The rise time (*g*) and decay time (*h*) of CH_3_NH_3_PbX_3_ detectors with varying voltage.

To measure response speed of the photodetectors, time-dependent response of CH_3_NH_3_PbX_3_ photodetectors under modulated illumination were measured as shown in figure 4*a*–*c*, showing good repeatability of our detectors. Photo-switching response under different voltage is given in figure 4*d*–*f*. The estimated rise/decay time versus voltage is displayed in figure 4*g*,*h*, respectively. As voltage increases, response time decreases originally and saturates finally. As we known, the response time *t* is decided by the carrier lifetime. As we mentioned above, the three detectors operate on photoconductivity mechanism, in which the trap states elongate the carrier lifetime. Thus, we speculate that the increased voltage weakens the trapping time of trap states on holes (electrons), which means that the carrier lifetimes are relatively decreased and then the response speed is decreased. As seen from table 2, the response times of CH_3_NH_3_PbX_3_ detectors are 10^1^–10^3^ times shorter than previously reported SrRuO_3_/BaTiO_3_/ZnO (7.1 s, 2.3 s) [40], β-Ga_2_O_3_ (3.33 s, 0.4 s) [45] and NaTaO_3_ (50 ms) [46].

**Table 2:** Comparison of response speed to deep-UV from several different semiconductors.

materials	light (nm)	bias (V)	rise time	decay time
CH_3_NH_3_PbCl_3_	255	10	15 ms	31 ms
CH_3_NH_3_PbBr_3_	255	10	2.5 ms	2.5 ms
CH_3_NH_3_PbI_3_	255	10	2 ms	2 ms
SrRuO_3_/BaTiO_3_/ZnO [40]	260	6	7.1 s	2.3 s
β-Ga_2_O_3_ [45]	236	20	3.33 s	0.4 s
NaTaO_3_ [46]	280	5	50 ms	50 ms

Among the three perovskite detectors, CH_3_NH_3_PbCl_3_ detector has the slowest response speed with rise time and rise time of 31 ms and 15 ms, respectively, which are 10 times longer than that of CH_3_NH_3_PbBr_3_ and CH_3_NH_3_PbI_3_ detectors, which have rise/decay time of about 2 ms. The slower response speed of CH_3_NH_3_PbCl_3_ detector can be attributed to the higher density of trap states in CH_3_NH_3_PbCl_3_ single crystals. This point is consistent with previous analysis on the responsivities.

Present research on CH_3_NH_3_PbX_3_ mainly focuses on polycrystalline-film-based solar cells, while their potential for deep-UV detection is not developed although they have outstanding optoelectronic properties suitable for deep-UV detection. Herein, we firstly give comprehensive studies on deep-UV detection performance of CH_3_NH_3_PbX_3_ (X = Cl, Br, I) single crystals.

To reveal the decisive role of intrinsic optoelectronic properties of perovskite on detector performance, high quality bulk crystals are used to fabricate planar-type MSM detectors, which operate on photoconductivity of CH_3_NH_3_PbX_3_. For such photoconductive detectors, there generally exists persistent photoconductivity mechanism. Trap states capture photo-generated carriers, and persistent photoconductivity (PPC) is formed, leading to an increased responsivity, simultaneously; the response speed is slowed.

From previous analysis on the dark current of the detectors shown in figure 3*a*–*c*, it is concluded that CH_3_NH_3_PbCl_3_ has highest density of traps among the three crystals. Therefore, according to the PPC mechanism, CH_3_NH_3_PbCl_3_ detector theoretically has the largest responsivity and slowest response speed, which is consistent with the measured results summarized in tables 1 and 2. CH_3_NH_3_PbBr_3_ and CH_3_NH_3_PbI_3_ crystals with low density of trap states have high responsivities and ultra-fast response speed, which seems more suitable for application in fast speed deep-UV detection.

## 4. Conclusion

In summary, we have grown millimetre-sized CH_3_NH_3_PbX_3_ (X = Cl, Br and I) bulk single crystals and used them to fabricate photodetectors. Benefiting from the excellent optoelectronic properties and high crystalline quality of CH_3_NH_3_PbX_3_ crystals, the detectors have low dark current, high photo/dark current ratio, sensitive and fast response speed to 255 nm deep-UV light. These excellent response performances make CH_3_NH_3_PbX_3_ materials promising candidates for deep-UV detection.

## Supplementary Material

Supporting information

## Supplementary Material

absorption

## Supplementary Material

photoluminescence

## Supplementary Material

XRD

## Supplementary Material

Photoresponse

## Data accessibility

The datasets supporting this article have been uploaded as part of the electronic supplementary material.

## References

[1] Boggess A et al. 1978 The IUE spacecraft and instrumentation. Nature 275, 372–377. (10.1038/275372a0)

[2] Xu Z, Ding H, Sadler BM, Chen G 2008 Analytical performance study of solar blind non-line-of-sight ultraviolet short-range communication links. Opt. Lett. 33, 1860–1862. (10.1364/OL.33.001860) 18709113

[3] Lin R, Zheng W, Zhang D, Zhang Z, Liao Q, Yang L, Huang F 2018 High-performance graphene/β-Ga**_2_**O**_3_** heterojunction deep-ultraviolet photodetector with hot-electron excited carrier multiplication. ACS Appl. Mater. Interfaces 10, 22 419–22 426. (10.1021/acsami.8b05336) 29897734

[4] Zheng W, Lin R, Zhang D, Jia L, Ji X, Huang F In press. Vacuum-ultraviolet photovoltaic detector with improved response speed and responsivity via heating annihilation trap states mechanism. Adv. Opt. Mater. (10.1002/adom.201800697)

[5] Zheng W, Lin R, Zhang Z, Huang F 2018 Vacuum-ultraviolet photodetection in few-layered h-BN. ACS Appl. Mater. Interfaces 10, 27 116–27 123. (10.1021/acsami.8b07189) 30043606

[6] Zheng W, Huang F, Zheng R, Wu H 2015 Low-dimensional structure vacuum-ultraviolet-sensitive (*λ* < 200 nm) photodetector with fast-response speed based on high-quality AlN micro/nanowire. Adv. Mater. 27, 3921–3927. (10.1002/adma.201500268) 26016601

[7] Schühle U, Hochedez J-F 2013 Solar-blind UV detectors based on wide band gap semiconductors, pp. 467–477. New York, NY: Springer.

[8] Mutlu G, Serkan B, Piotr C, Wlodek S, Ekmel O 2012 Integrated AlGaN quadruple-band ultraviolet photodetectors. Semicond. Sci. Technol. 27 065004 (10.1088/0268-1242/27/6/0650)

[9] Ju ZG, Shan CX, Jiang DY, Zhang JY, Yao B, Zhao DX, Shen DZ, Fan XW 2008 Mg**_x_**Zn**_1−x_**O-based photodetectors covering the whole solar-blind spectrum range. Appl. Phys. Lett. 93, 173505 (10.1063/1.3002371)

[10] Mendoza F, Makarov V, Weiner BR, Morell G 2015 Solar-blind field-emission diamond ultraviolet detector. Appl. Phys. Lett. 107, 5 (10.1063/1.4936162)

[11] Hongyu C, Hui L, Zhiming Z, Kai H, Xiaosheng F 2016 Nanostructured photodetectors: from ultraviolet to terahertz. Adv. Mater. 28, 403–433. (10.1002/adma.201503534) 26601617

[12] Zhang D, Zheng W, Lin RC, Li TT, Zhang ZJ, Huang F 2018 High quality β-Ga**_2_**O**_3_** film grown with N**_2_**O for high sensitivity solar-blind-ultraviolet photodetector with fast response speed. J. Alloys Compd. 735, 150–154. (10.1016/j.jallcom.2017.11.037)

[13] Zhang D, Zheng W, Zheng Q, Chen A, Ji X, Huang F 2016 A strategy of transparent conductive oxide for UV focal plane array detector: two-step thermodynamic process. Adv. Electron. Mater. 2, 1600320 (10.1002/aelm.201600320)

[14] Li W-D, Chou SY 2010 Solar-blind deep-UV band-pass filter (250–350 nm) consisting of a metal nano-grid fabricated by nanoimprint lithography. Opt. Express 18, 931–937. (10.1364/OE.18.000931) 20173915

[15] Hennessy J, Jewell AD, Hoenk ME, Nikzad S 2015 Metal-dielectric filters for solar-blind silicon ultraviolet detectors. Appl. Opt. 54, 3507–3512. (10.1364/ao.54.003507) 25967344

[16] Heo JH, Song DH, Im SH 2014 Planar CH**_3_**NH**_3_**PbBr**_3_** hybrid solar cells with 10.4% power conversion efficiency, fabricated by controlled crystallization in the spin-coating process. Adv. Mater. 26, 8179–8183. (10.1002/adma.201403140) 25348285

[17] Green MA, Ho-Baillie A, Snaith HJ 2014 The emergence of perovskite solar cells. Nat. Photonics 8, 506–514. (10.1038/nphoton.2014.134)

[18] Dong Q, Fang Y, Shao Y, Mulligan P, Qiu J, Cao L, Huang J 2015 Electron-hole diffusion lengths > 175 μm in solution-grown CH_3_NH_3_PbI_3_ single crystals. Science 347, 967–970. (10.1126/science.aaa5760) 25636799

[19] Zheng W, Lin R, Zhang Z, Liao Q, Liu J, Huang F 2017 An ultrafast-temporally-responsive flexible photodetector with high sensitivity based on high-crystallinity organic–inorganic perovskite nanoflake. Nanoscale 9, 12 718–12 726. (10.1039/C7NR04395C) 28829096

[20] Maculan G et al. 2015 CH**_3_**NH**_3_**PbCl**_3_** single crystals: inverse temperature crystallization and visible-blind UV-photodetector. J. Phys. Chem. Lett. 6, 3781–3786. (10.1021/acs.jpclett.5b01666) 26722870

[21] Saidaminov MI et al. 2015 High-quality bulk hybrid perovskite single crystals within minutes by inverse temperature crystallization. Nat. Commun. 6, 7586 (10.1038/ncomms8586) 26145157 PMC4544059

[22] Liu Y et al. 2015 Two-inch-sized perovskite CH**_3_**NH**_3_**PbX**_3_** (X = Cl, Br, I) crystals: growth and characterization. Adv. Mater. 27, 5176–5183. (10.1002/adma.201502597) 26247401

[23] Tanaka K, Takahashi T, Ban T, Kondo T, Uchida K, Miura N 2003 Comparative study on the excitons in lead-halide-based perovskite-type crystals CH**_3_**NH**_3_**PbBr**_3_** CH**_3_**NH**_3_**PbI**_3_**. Solid State Commun. 127, 619–623. (10.1016/S0038-1098(03)00566-0)

[24] Kitazawa N, Watanabe Y, Nakamura Y 2002 Optical properties of CH**_3_**NH**_3_**PbX**_3_** (X = halogen) and their mixed-halide crystals. J. Mater. Sci. 37, 3585–3587. (10.1023/a:1016584519829)

[25] Stoumpos CC, Malliakas CD, Kanatzidis MG 2013 Semiconducting tin and lead iodide perovskites with organic cations: phase transitions, high mobilities, and near-infrared photoluminescent properties. Inorg. Chem. 52, 9019–9038. (10.1021/ic401215x) 23834108

[26] Shi D *et al* 2015 Low trap-state density and long carrier diffusion in organolead trihalide perovskite single crystals. Science 347, 519–522. (10.1126/science.aaa2725) 25635092

[27] Fang Y, Dong Q, Shao Y, Yuan Y, Huang J 2015 Highly narrowband perovskite single-crystal photodetectors enabled by surface-charge recombination. Nat. Photonics 9, 679–686. (10.1038/nphoton.2015.156)

[28] Wei H et al. 2016 Sensitive X-ray detectors made of methylammonium lead tribromide perovskite single crystals. Nat. Photonics 10, 333–339. (10.1038/nphoton.2016.41)

[29] Stoumpos CC, Kanatzidis MG 2015 The renaissance of halide perovskites and their evolution as emerging semiconductors. Acc. Chem. Res. 48, 2791–2802. (10.1021/acs.accounts.5b00229) 26350149

[30] Tauc J, Grigorovici R, Vancu A 1966 Optical properties and electronic structure of amorphous germanium. Phys. Status Solidi (b) 15, 627–637. (10.1002/pssb.19660150224)

[31] Davis EA, Mott NF 1970 Conduction in non-crystalline systems V. Conductivity, optical absorption and photoconductivity in amorphous semiconductors. Philos. Mag.: A Journal of Theoretical Experimental and Applied Physics 22, 0903–0922. (10.1080/14786437008221061)

[32] Huang LY, Lambrecht WRL 2013 Electronic band structure, phonons, and exciton binding energies of halide perovskites CsSnCl**_3_**, CsSnBr**_3_**, and CsSnI**_3_**. Phys. Rev. B, 88, 165203 (10.1103/PhysRevB.88.165203)

[33] Poglitsch A, Weber D 1987 Dynamic disorder in methylammoniumtrihalogenoplumbates (II) observed by millimeter-wave spectroscopy. J. Chem. Phys. 87, 6373–6378. (10.1063/1.453467)

[34] Zheng W, Lin R, Zhu Y, Zhang Z, Ji X, Huang F 2018 Vacuum-ultraviolet photodetection in two-dimensional oxides. ACS Appl. Mater. Interfaces 10, 20 696–20 702. (10.1021/acsami.8b04866) 29808671

[35] Zhang Z, Zhu Y, Wang W, Zheng W, Lin R, Huang F 2018 Growth, characterization and optoelectronic applications of pure-phase large-area CsPb**_2_**Br**_5_** flake single crystals. J. Mater. Chem. C 6, 446–451. (10.1039/C7TC04834C)

[36] Zhang Z, Zheng W, Chen A, Ding K, Huang F 2015 Crystal growth of α-HgI**_2_** by the temperature difference method for high sensitivity X-ray detection. Cryst. Growth Des. 15, 3383–3387. (10.1021/acs.cgd.5b00468)

[37] Zheng W, Xiong X, Lin R, Zhang Z, Xu C, Huang F 2018 Balanced photodetection in one-step liquid-phase-synthesized CsPbBr**_3_** micro-/nanoflake single crystals. ACS Appl. Mater. Interfaces 10, 1865–1870. (10.1021/acsami.7b18093) 29265802

[38] Konstantatos G, Clifford J, Levina L, Sargent EH 2007 Sensitive solution-processed visible-wavelength photodetectors. Nat. Photonics 1, 531 (10.1038/nphoton.2007.147)

[39] Wei TC, Tsai DS, Ravadgar P, Ke JJ, Tsai ML, Lien DH, Huang CY, Horng RH, He JH 2014 See-through Ga**_2_**O**_3_** solar-blind photodetectors for use in harsh environments. IEEE J. Sel. Top. Quantum. Electron. 20, 1–6. (10.1109/JSTQE.2014.2321517)

[40] Pandey BK, Dias S, Nanda KK, Krupanidhi SB 2017 Deep UV-Vis photodetector based on ferroelectric/semiconductor heterojunction. J. Appl. Phys. 122, 234502 (10.1063/1.4994780)

[41] Bin Z, Fei W, Hongyu C, Lingxia Z, Longxing S, Dongxu Z, Xiaosheng F 2017 An ultrahigh responsivity (9.7 mA W^−1^) self-powered solar-blind photodetector based on individual ZnO–Ga**_2_**O**_3_** heterostructures. Adv. Funct. Mater. 27, 1700264 (10.1002/adfm.201700264)

[42] Hongyu C, Pingping Y, Zhenzhong Z, Feng T, Lingxia Z, Kai H, Xiaosheng F 2016 Ultrasensitive self-powered solar-blind deep-ultraviolet photodetector based on all-solid-state polyaniline/MgZnO bilayer. Small 12, 5809–5816. (10.1002/smll.201601913) 27594337

[43] Xing J, Guo E, Jin K-J, Lu H, Wen J, Yang G 2009 Solar-blind deep-ultraviolet photodetectors based on an LaAlO_3_ single crystal. Opt. Lett. 34, 1675–1677. (10.1364/OL.34.001675) 19488145

[44] Zheng W, Lin R, Ran J, Zhang Z, Ji X, Huang F 2018 Vacuum-ultraviolet photovoltaic detector. ACS Nano 12, 425–431. (10.1021/acsnano.7b06633) 29298035

[45] Pratiyush AS, Krishnamoorthy S, Solanke SV, Xia Z, Muralidharan R, Rajan S, Nath DN 2017 High responsivity in molecular beam epitaxy grown β-Ga**_2_**O**_3_** metal semiconductor metal solar blind deep-UV photodetector. Appl. Phys. Lett. 110, 221107 (10.1063/1.4984904)

[46] Guo B, Wu G, Chen H, Wang M 2015 Two-step hydrothermal synthesis of sodium tantalate nanoparticles with deep ultraviolet sensitivity. J. Mater. Chem. C 3, 9346–9352. (10.1039/C5TC02334C)

