# Supplementary material for: High-sensitive and fast response to 255 nm deep-UV light of CH3NH3PbX3 (X = Cl, Br, I) bulk crystals
Source: R Soc Open Sci. 2018 Sep 5;5(9):180905. doi: 10.1098/rsos.180905 (PMC6170544; doi:10.1098/rsos.180905)
Supplement: Supporting information [file rsos180905supp1.docx]

**Supporting information**

**Crystal Characterization.**

The powder X-ray diffraction (XRD) patterns were collected using a Bruker D8 Advance X-ray diffractometer operated at 40 kV and 40 mA. UV-VIS-NIR diffuse reflectance spectra were collected using the Perkin Elmer Lambda 950 spectrophotometer equipped with integrating sphere. Barium sulfate (BaSO_4_) powder plate was employed as the standard reference (100% reflectance) when measuring the reflectance spectra of the CH_3_NH_3_PbX_3_ powder. Photoluminescence spectra was measured at room temperature using a Jobin Yvon LabRAM HR Raman spectrometer with using a 325 nm laser as excitation source.

**Photodetector fabrication and photo-response testing.**

Au films were coated on the crystal surface as electrode by thermal evaporation. The photo-response were measured using self-built system. 255 nm LED was employed as light source, Keithley 2636B source meter was used to measure the I-V traces of the fabricated photodetectors.
